# Informiert = Geimpft? Das Informationsverhalten und die COVID-19-Impfentscheidung bei Studierenden

**DOI:** 10.1007/s11616-023-00779-7

**Published:** 2023-01-26

**Authors:** Markus Schäfer, Birgit Stark, Antonia M. Werner, Lina M. Mülder, Jennifer L. Reichel, Sebastian Heller, Lisa Schwab, Thomas Rigotti, Manfred E. Beutel, Perikles Simon, Stephan Letzel, Pavel Dietz

**Affiliations:** 1 Institut für Publizistik, Johannes Gutenberg-Universität Mainz, Jakob-Welder-Weg 12, 55128 Mainz, Deutschland; 2 Klinik und Poliklinik für Psychosomatische Medizin und Psychotherapie, Universitätsmedizin, Johannes Gutenberg-Universität Mainz, Untere Zahlbacher Str. 8, 55131 Mainz, Deutschland; 3 Arbeits‑, Organisations- und Wirtschaftspsychologie, Institut für Psychologie, Johannes Gutenberg-Universität Mainz, Wallstraße 3, 55122 Mainz, Deutschland; 4 Institut für Arbeits‑, Sozial- und Umweltmedizin, Universitätsmedizin, Johannes Gutenberg-Universität Mainz, Obere Zahlbacher Str. 67, 55131 Mainz, Deutschland; 5 Institut für Sportwissenschaft, Johannes Gutenberg-Universität Mainz, Albert Schweitzer Straße 22, 55128 Mainz, Deutschland; 6 Leibniz Institut für Resilienzforschung, Wallstr. 7a, 55122 Mainz, Deutschland

**Keywords:** Informationsverhalten, Mediennutzung, Medienvertrauen, COVID-19, Impfung, Information seeking, Media use, Media trust, COVID-19, Vaccination

## Abstract

Eine zu geringe Impfbereitschaft zählt zu den größten globalen Gesundheitsgefahren und war in der COVID-19-Pandemie auch in Deutschland eine der großen Herausforderungen der öffentlichen Gesundheit. Die Identifikation potenzieller Einflussfaktoren auf das Impfverhalten ist deshalb für eine zielgruppengerechte Gesundheitskommunikation von großer Bedeutung. Studierende sind eine besonders wichtige Zielgruppe der Prävention und Gesundheitsförderung. Der Beitrag geht mit Hilfe einer Online-Befragung der Studierenden einer westdeutschen Universität (*n* = 1398) im Sommersemester 2021 den Fragen nach, inwieweit sich geimpfte und ungeimpfte Studierende mit hoher bzw. niedrigerer Impfintention hinsichtlich a) ihrer Medien- und Informationsnutzung und b) ihres Vertrauens in Medien und Informationsquellen in der COVID-19-Pandemie unterschieden. Die Ergebnisse zeigen z. T. deutliche Differenzen. Während geimpfte Studierende sich intensiver informierten und hierfür auch stärker auf klassische Medienangebote zurückgriifen, vertrauten insbesondere ungeimpfte Studierende mit niedrigerer Impfintention u. a. mehr auf alternative Nachrichtenseiten und Blogs.

## Abstract (en)

Vaccinations prevent several million deaths every year and have saved many lives in the COVID-19 pandemic. At the same time, vaccination hesitancy is one of the biggest global health threats and has been one of the major public health challenges during the pandemic in Germany. The identification of influencing factors on vaccination attitudes and behavior is especially important for targeted and tailored communication. Use of and trust in mass media and (other) information channels are among the potential factors that are regularly discussed in this context. Some international findings suggested that the use of traditional mass media could be associated with more realistic perceptions of vaccination and its benefits and risks and a higher intention to get vaccinated, while increased exposure to vaccination information on social media channels could be associated with increased false knowledge and increase vaccination hesitancy.

The vaccination behavior of university students in the COVID-19 pandemic is of particular scientific and social interest. University students and their living and working environments at colleges and universities are generally considered to be particularly important target points for prevention and health promotion. Although their specific social and health situation has long been neglected by German policymakers during COVID-19 pandemic, university students as a young, active, and mobile subpopulation have repeatedly come into focus of pandemic containment and international research. International surveys in European countries such as France, Italy, Belgium, the Netherlands, Portugal, or the Czech Republic reported a (hypothetical) willingness to vaccinate among university students between 58 and 86% for COVID-19 vaccination, indicating that relevant proportions among the students were hesitant whether to get vaccinated against COVID-19 or not.

Using the example of a German university in the summer term of 2021 and based on an online survey of university students (*n* = 1398), the present paper investigates to what extent vaccinated and unvaccinated students with high and (rather) low intention to get vaccinated differed a) with regard to their media and information use and b) their trust in media and information sources during COVID-19 pandemic. The online survey took place between June 21 and August 15, 2021, a few weeks after prioritization for COVID-19 vaccines ended. All students at the university were contacted via a central mailing list, through which they usually receive important news, for example about their grades. 1398 questionnaires in which the vaccination status was clearly stated were considered for analysis. Among the participants in our sample, younger and female students were overrepresented, compared to the distribution of these characteristics at the university respective among university students in Germany. More than two-thirds of the students had already received at least one vaccination against COVID-19 in the summer semester of 2021 (69.7%), just under one-third (30.3%) had not yet been vaccinated at that time. Vaccination readiness among unvaccinated students proved to be high, with nearly two-thirds (64.3%) reporting that they were “very likely” to be vaccinated against COVID-19.

Using ANOVA and chi-square-tests, we systematically explored differences and similarities between vaccinated and unvaccinated students with high and low vaccination intention. The results showed significant differences between the three groups regarding their COVID-19-related information seeking and their trust in media and information sources. Vaccinated students used topic-related information more intensively than unvaccinated students with high vaccination intention and low vaccination intention, noticed that the latter informed themselves even less intensively than the students with high intention. Unvaccinated students were less likely to use offline media, in general and as topic-related source of information and were less likely to obtain corona-related information from health care professionals. A significantly lower proportion of unvaccinated students with a lower intention to get vaccinated used the official COVID-19 warning app and official websites of governments and public authorities as information sources. Students without COVID-19 vaccination and lower vaccination intention further expressed lower trust in the federal, state, and city government, and health authorities as information sources, whereas they rated alternative news sites and blogs as more trustworthy than their vaccinated fellow students.

Our study provides important insights into university students’ vaccination behavior and possible communication-related influencing factors, which could especially be useful in the design of suitable and tailored target-group-specific campaigns to reduce vaccination hesitancy. To support and empower students during their primarily online-based topic-related information seeking, it may make sense not only to implement specific measures to prevent the dissemination of false information, but also to strengthen students’ media and health literacy with targeted programs to enable them to distinguish between reputable and dubious providers and sources, regardless of the channels they use, and to make appropriate health and vaccination decisions. When communicating vaccination-related information, it could further make sense to focus more on channels and actors that the rather sceptical members of the target group have comparatively high confidence in, given their absolute level of trust. As our survey showed comparably high levels of trust for health professionals and universities as information sources, in order to better reach vaccine-skeptical students, it could be helpful to use this knowledge and to reduce barriers and get those students better into conversation with health professionals. At the same time, it seems important to include colleges and universities as working and living environments of students even more in vaccination campaigns.

## Einleitung

Kommunikation ist ein zentraler Schlüssel für den Erfolg von Impfkampagnen (vgl. Metz 2022). Während Wissenschaftler:innen und Vertreter:innen von Gesundheitsbehörden in der COVID-19-Pandemie in traditionellen Massenmedien die Vorteile einer Impfung betonten, wurden randständige Ansichten über den Nutzen von COVID-19-Impfungen häufig über alternative oder soziale Medien verbreitet. Vor allem Impfkritiker vernetzten sich auf Plattformen wie Twitter oder Telegram und beeinflussten öffentliche (Impf‑)Debatten und damit auch individuelle Meinungsbildungsprozesse (vgl. z. B. Hajek 2022; Theocharis et al. 2021). Dabei gelten Impfungen als eine wichtige Errungenschaft der modernen Medizin, die jedes Jahr mehrere Millionen Todesfälle verhindert und auch in der COVID-19-Pandemie schon viele Leben gerettet hat (vgl. UNICEF 2021; WHO-Regionalbüro für Europa 2021). Auch deshalb gehörte für die Weltgesundheitsorganisation WHO (2020) in der Pandemie der schnelle Zugang zu wirksamen Impfstoffen zu den zehn wichtigsten Aspekten der globalen Gesundheit. Gleichzeitig zählte die WHO (2019) schon vor der Pandemie eine zu geringe Impfbereitschaft bzw. zu hohe Impfmüdigkeit zu den zehn größten globalen Gesundheitsgefahren.^1^

Impfbereitschaft und Impfmüdigkeit unterliegen im Allgemeinen einer Vielzahl potenzieller Einflussfaktoren auf Mikro‑, Meso- und Makroebene (vgl. Hildt et al. 2019, S. 383; MacDonald 2015, S. 4163; Shmueli 2021, S. 2–3). Welche Rolle das *Informationsverhalten* spielt, ist bislang in Ansätzen untersucht (vgl. z. B. Betsch et al. 2010; Nan und Madden 2012; Rubin et al. 2010; Stecula et al. 2020). Hinweise gab es u. a. darauf, dass die Nutzung traditioneller Massenmedien mit realistischeren Vorstellungen von Impfungen und deren Nutzen und Risiken einhergehen könnte, während etwa ein häufigerer Kontakt mit Impfinformationen auf Social Media-Kanälen verstärkt mit falschem Wissen assoziiert wurde (vgl. Stecula et al. 2020, S. 4–7).

Auch bei der COVID-19-Impfentscheidung deutet vieles darauf hin, dass das Informationsverhalten ein relevanter Einflussfaktor sein könnte (vgl. Gehrau et al. 2021; Lazarus et al. 2021; Murphy et al. 2021; Zimand-Sheiner et al. 2021). Vor allem die konkrete *Medien- und Informationsnutzung* und hier insbesondere die Rezeption von Falschinformationen und Impfmythen, die in Kombination mit fehlendem Wissen und fehlender Medien- und Gesundheitskompetenz bei Rezipient:innen Zweifel an der Sicherheit und Wirksamkeit von Impfungen säen könnten, wurden hier zuletzt regelmäßig als wichtige Faktoren diskutiert (vgl. Europäische Kommission 2021; Geiger et al. 2021; Lewandowsky et al. 2021; Puri et al. 2020). Gerade in einer Pandemie mit oftmals unklarer oder mehrdeutiger Informationslage könnte zudem das *Vertrauen* der Rezipient:innen in (bestimmte) Informationsquellen und Medienkanäle von großer Bedeutung sein (vgl. Gehrau et al. 2021, S. 2; Larson et al. 2018, S. 1600; Murphy et al. 2021, S. 4; Zimand-Sheiner et al. 2021, S. 10).

Dies gilt umso mehr, als weitgehend Konsens darüber besteht, dass Vertrauen auf der einen (vgl. Eitze et al. 2021, S. 268; Koos 2021, S. 6) und die Medien- und Informationsnutzung auf der anderen Seite (vgl. Collinson et al. 2015, S. 14; Cruwys et al. 2020, S. 587–588; Friemel und Geber 2021, S. 4; Koos 2021, S. 6; Rubin et al. 2010, S. 232; Schäfer et al. 2021, S. 8; Shore 2003, S. 13) für gesundheitsbezogene Vorstellungen, Einstellungen und Verhalten (in Pandemien) einflussreich sind. Die Forschung liefert darüber hinaus Hinweise darauf, dass zwischen dem Vertrauen der „Vertrauensgeber“ in verschiedene Objekte (wie z. B. andere Menschen, politische Institutionen, Medien, das Gesundheitssystem, aber eben auch Impfungen) systematische Zusammenhänge bestehen (vgl. Fawzi et al. 2021, S. 158–159; Haug et al. 2021, S. 791; Seddig et al. 2021, S. 10; Tsfati und Ariely 2014, S. 770). Auch ist bekannt, dass die Medien- und Informationsnutzung mit dem Vertrauen interagieren kann (vgl. Fawzi et al. 2021, S. 160–163; Jakobs et al. 2021, S. 476–480; Newman et al. 2021, S. 11; Quiring et al. 2021, S. 3510; Tsfati und Ariely 2014, S. 769–770; Viehmann et al. 2020, S. 566). Der erste Punkt deutet darauf hin, dass Vertrauen (in Institutionen) einen starken habituellen bzw. generalisierten Anteil haben kann, der zweite Punkt insbesondere darauf, dass kommunikative Aspekte bei der Entstehung und Kultivierung von Vertrauen von Bedeutung sind. Gleichzeitig könnten Vertrauensaspekte für die Intensität und die Art der Medien- und Informationsnutzung relevant sein.

Auch beim Thema Impfen mehren sich die Indizien, dass das Vertrauen in bestimmte Informationskanäle und die konkrete Informations- und Mediennutzung Vorstellungen, Impfeinstellungen und Impfentscheidungen beeinflussen können (vgl. Betsch et al. 2010, S. 446, 2019, S. 401; Gehrau et al. 2021, S. 2; Taha et al. 2013; Wismans et al. 2021, S. 9). Allerdings ist der empirische Forschungsstand zur Rolle des Informationsverhaltens gerade mit Blick auf Deutschland und die COVID-19-Impfung bislang unbefriedigend. Die vorliegende Studie verfolgt deshalb das Ziel, mit einer Online-Befragung unter Studierenden der Rolle kommunikationsbezogener Faktoren bei der Impfentscheidung nachzugehen.

Dabei fragen wir, inwieweit sich geimpfte Studierende, ungeimpfte Studierende mit hoher Impfintention und niedrigerer Impfintention hinsichtlich a) ihrer Medien- und Informationsnutzung und b) ihres Vertrauens in Medien und Informationsquellen in der COVID-19-Pandemie unterscheiden. Um diese Fragen zu beantworten, stützt sich der Beitrag auf eine Online-Befragung der Studierenden der Johannes Gutenberg-Universität Mainz (*n* = 1398) im Sommersemester 2021 nach Aufhebung der Impfpriorisierung für die COVID-19-Impfstoffe.

Das Impfverhalten von Studierenden in der COVID-19-Pandemie ist von besonderem wissenschaftlichem wie gesellschaftlichem Interesse. Studierende und ihre Lebens- und Arbeitswelt der Hochschulen und Universitäten gelten allgemein als besonders wichtige Zielpunkte der Prävention und Gesundheitsförderung (vgl. Barello et al. 2020, S. 781; Dietz et al. 2020; Schäfer et al. 2019). Auch mit Blick auf die Eindämmung der COVID-19-Pandemie sind Studierende als junge, aktive und mobile Teilpopulation immer wieder ins Zentrum der Aufmerksamkeit geraten (vgl. Barello et al. 2020; Ioannidis 2021; Rossmann et al. 2021; Schäfer et al. 2021; Wismans et al. 2020, 2021), wenngleich ihre soziale und gesundheitliche Situation in Deutschland von der Politik lange Zeit vernachlässigt wurde (vgl. Dietz et al. 2021). Studierendenbefragungen in Ländern wie Frankreich, Italien, Belgien, den Niederlanden, Portugal oder Tschechien fanden zudem zwischen Frühjahr 2020 und Frühsommer 2021 für die COVID-19-Impfung eine hypothetische Impfbereitschaft unter den befragten Studierenden zwischen 58 und 86 % (vgl. Barello et al. 2020, S. 781; Riad et al. 2021, S. 7; Tavolacci et al. 2021, S. 5; Wismans et al. 2021, S. 10). Das macht deutlich, dass auch unter europäischen Studierenden relevante Teile einer COVID-19 Impfung skeptisch oder zögerlich gegenüberstanden. Faktoren zu untersuchen, die eine Impfbereitschaft bzw. Impfzögerlichkeit bei Studierenden begünstigen, ist daher auch für Deutschland sinnvoll.

## Einflussfaktoren beim Impfen: Die Rolle des Informationsverhaltens

### Allgemeine empirische Befunde und theoretische Überlegungen

Impfbereitschaft und Impfmüdigkeit sind keine stabilen Eigenschaften, sondern kontextspezifische Zustände auf einem Kontinuum, die u. a. in Abhängigkeit von der Art der Impfung und des Impfstoffs, der Zeit, dem Ort usw. variieren können (vgl. Betsch et al. 2019, S. 400; Geiger et al. 2021). Hinzu kommt, dass das konkrete Impfverhalten nicht nur von motivationalen Faktoren, sondern z. B. auch vom individuellen Zugang, den verfügbaren Zeitressourcen, dem rechtlichen Rahmen oder schlicht dem Erinnern oder Vergessen eines Impftermins abhängen kann (vgl. Betsch et al. 2019, S. 400; Geiger et al. 2021; Horstkötter et al. 2017, S. 66, 2019, S. 66, 2021, S. 68). Mögliche Einflussfaktoren auf Impfintentionen und Impfverhalten waren schon lange vor der COVID-19-Pandemie Gegenstand wissenschaftlicher Untersuchungen (für einen Überblick vgl. Betsch et al. 2018, S. 2–4; MacDonald 2015, S. 4162–4163). Zu diesen potenziellen Einflussfaktoren zählen auf individueller Ebene soziodemographische Faktoren wie Alter, Geschlecht oder Bildung, gesundheitsbezogene Parameter wie das Vorliegen chronischer Erkrankungen, psychologische Faktoren wie das Vertrauen in die Sicherheit und Effektivität von Impfungen oder die Wahrnehmung von Risiken, Normen und Barrieren, aber auch externe Einflussfaktoren wie rechtliche Regelungen oder die Qualität der Impfkommunikation (vgl. Bish et al. 2011; Graupensperger et al. 2021; Schmid et al. 2017). Beobachtbar sind zudem nicht selten regionale Unterschiede, sowohl zwischen Ländern als auch innerhalb eines Landes (vgl. De Figueiredo et al. 2020; Dubé et al. 2014; Horstkötter et al. 2017, 2019).

Dass sich auch das *Informationsverhalten* unter Umständen (und auch bei COVID-19) mittelbar oder unmittelbar auf die Impfentscheidung auswirkt, ist durchaus plausibel, zumal bekannt ist, dass Medien für viele Menschen gerade in Pandemien wichtige und einflussreiche Informationsquellen darstellen (vgl. Baumann et al. 2020; Schäfer 2020; Taha et al. 2013; Viehmann et al. 2020, 2021; Zhao und Wu 2021). In der Vergangenheit haben sich kommunikationsbezogene Faktoren auch für gesundheitliches Handeln als wichtige Einflussgrößen erwiesen (vgl. u. a. Binder et al. 2014; Carpenter 2010; Friemel und Geber 2021; Green et al. 2021; Rosenstock et al. 1988). Von besonderer Bedeutung sind dabei die *Medien- und Informationsnutzung* sowie das *Vertrauen* in bestimmte Medien- und Informationsquellen (vgl. Gehrau et al. 2021).

Verschiedene Autor:innen konzipieren die Medien- und Informationsnutzung und das Quellenvertrauen implizit oder explizit als wichtige Einflussgrößen in allgemeineren Modellen des gesundheitsbezogenen Informationshandelns. Das „Risk Information Seeking and Processing Model“ (RISP, vgl. Griffin et al. 1999, 2013) etwa geht davon aus, dass sich die Vorstellungen von der Vertrauenswürdigkeit der Informationskanäle auf die Informationssuche und -verarbeitungsstrategien der Rezipient:innen auswirken. So würden etwa Informationen aus Medien aktiver verarbeitet, die in den Vorstellungen der Rezipient:innen eine schlechtere Qualität aufweisen und gegenüber deren Inhalten die Rezipient:innen negativer eingestellt sind. In ihrer Weiterentwicklung des „Planned Risk Information Seeking Models“ (PRISM, vgl. Kahlor 2010) nahmen Hovick et al. (2014) an, dass vergangene Sucherfahrungen u. a. die Vorstellungen davon prägen, inwieweit Quellen vertrauenswürdig sind. Diese Vorstellungen wiederum hätten Einfluss auf die Einstellungen zur Informationssuche, die sich ihrerseits auf die Suchintentionen auswirkten. Tatsächlich erwiesen sich die Quellenvorstellungen in einer empirischen Überprüfung des Modells bei der Suche nach Informationen zum eigenen Krebsrisiko als bedeutende Einflussfaktoren. Link (2019) erweiterte das PRISM explizit um Vertrauenseinstellungen. In ihrer empirischen Studie zeigte sich u. a., dass ein höheres Vertrauen in ärztliche Informationen mit einem geringeren Vertrauen in Gesundheitsinformationen aus dem Internet einherging. Die Vertrauenseinstellungen gegenüber Online-Gesundheitsinformationen erwiesen sich bei Gesunden wie bei Erkrankten als wichtig für die Informationssuche im Internet. Auch bei der Anwendung des „Extended Parallel Process Model“ (EPPM, vgl. Witte 1994), das im Bereich Public Health häufig zum Einsatz kommt, um zu erklären, wie die Risikowahrnehmung Gesundheitsverhalten beeinflussen kann, werden der Kontakt mit bestimmten Kanälen und Informationen und das Quellenvertrauen regelmäßig als relevante Einflussgrößen modelliert, die sich auf die Wahrnehmungsprozesse auswirken – auch im Hinblick auf Gesundheitsverhalten in der COVID-19-Pandemie (vgl. u. a. Vaala et al. 2022; Zhao und Wu 2021).

**Figure 1:**
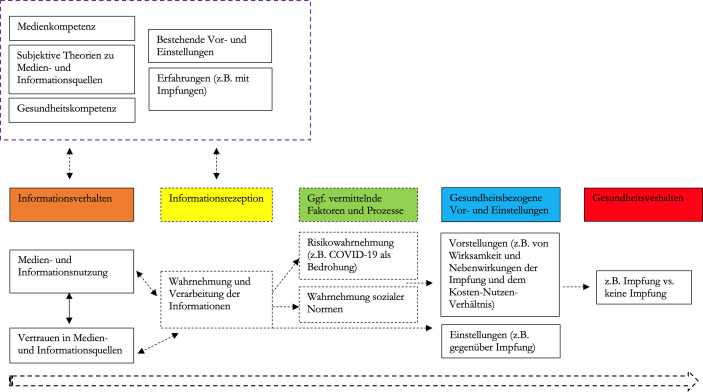

Vor dem Hintergrund dieser theoretischen Annahmen und der empirischen Befunde ist anzunehmen, dass das Vertrauen in Informationsquellen und die Mediennutzung in enger Beziehung stehen und maßgeblich darüber mitbestimmen, welche Informationen zum Thema Impfen wie rezipiert werden. Sie dürften sich so unmittelbar oder mittelbar auf gesundheitsbezogene Vorstellungen, Gesundheitseinstellungen und Gesundheitsverhalten auswirken (Abb. 1). Während das Quellenvertrauen nicht zuletzt darüber mitentscheidet, welche Kanäle überhaupt genutzt werden und wie Inhalte einzuordnen sind, bestimmt die Medien- und Informationsnutzung, mit welchen konkreten Informationen, Verhaltensmodellen (vgl. Bandura 2001) und Bewertungen oder Normen Rezipient:innen in Kontakt kommen. Im Zusammenspiel mit weiteren Faktoren und Prozessen leistet das Informationsverhalten somit einen grundlegenden Beitrag beim Erwerb bzw. der Modifikation von gesundheitsbezogenen Vorstellungen, Einstellungen und Verhaltensintentionen. Zudem ist denkbar, dass der Kontakt mit bestimmten Medieninhalten selbst als Auslöser für ein bestimmtes Gesundheitsverhalten fungiert (vgl. Bandura 2001) – und somit auch mehr oder weniger unmittelbare Wirkungen auf das Gesundheitsverhalten möglich sind.

Zu berücksichtigen ist dabei, dass Informationsverhalten und Informationsrezeption (natürlich auch das Gesundheitsverhalten) mit einer Vielzahl weiterer Faktoren interagieren (Abb. 1). So können etwa Medien- und Gesundheitskompetenz Informationsnutzung und Informationsrezeption beeinflussen (z. B. bei der Wahl der Kanäle oder der Einordnung der gefundenen Informationen; vgl. Bosle et al. 2022; Hugger 2021; Schäfer 2021). Subjektive Einstellungen zu Medien- und Informationsquellen (vgl. z. B. Palmer et al. 2020) können das Vertrauen in diese Quellen und die Interpretation der dort rezipierten Inhalte prägen. Bei der Wahrnehmung und Verarbeitung der Informationen dürften zudem auch bestehende Vorstellungen (z. B. über Gesundheitssystem, Ärzt:innen, Politik, Pharmaindustrie usw.) und eigene Erfahrungen (z. B. mit früheren Impfungen) darüber mitbestimmen, wie Medieninformationen wahrgenommen und interpretiert werden.

In diesem Beitrag liegt der Schwerpunkt nicht auf den Faktoren und Prozessen, die hinter dem Informationsverhalten stehen. Auch die Informationsrezeption und etwaige weitere Faktoren und Prozesse, die zwischen Informations- und Impfverhalten vermitteln, müssen hier ausgeklammert werden. Stattdessen lenken wir den Blick auf die Medien- und Informationsnutzung und das Vertrauen in Medien- und Informationsquellen und widmen uns der Frage, inwieweit sich Menschen, bei denen Unterschiede im Impfverhalten zu beobachten sind, in ihrem Informationsverhalten unterscheiden.

### Medien- und Informationsnutzung

Die Medien- und Informationsnutzung wird allgemein sowohl hinsichtlich ihrer *Intensität* als auch hinsichtlich der *Art der genutzten Kanäle und Quellen* als wichtiger Einflussfaktor für Impfintentionen und Impfverhalten diskutiert (vgl. Allington et al. 2021; Betsch et al. 2010, S. 446; Garfin et al. 2020; Gehrau et al. 2021, S. 2; Karlsson et al. 2021, S. 9; Murphy et al. 2021, S. 4). In Deutschland zeigte sich in repräsentativen Befragungen, dass sowohl massenmediale und interpersonale Quellen als auch Social Media beim Thema Impfen als Informationsquellen eine relevante Rolle spielen (vgl. Horstkötter et al. 2017, 2019, 2021). Besonders bemerkenswert ist dabei, dass das Internet und speziell soziale Medien sowohl vor als auch während der Pandemie insbesondere von den für Studierende maßgeblichen Altersgruppen der 16- bis 20- bzw. der 21- bis 29-Jährigen überproportional stark als geeignete Informationsquellen zum Thema Impfen bewertet wurden (vgl. Horstkötter et al. 2017, S. 119, 2019, S. 117, 2021, S. 121).

Darüber hinaus zeigen internationale Forschungen, dass die Art der genutzten Quellen mit der Impfbereitschaft in Verbindung stehen kann. Eine US-amerikanische Studie fand in einer bevölkerungsrepräsentativen Panel-Untersuchung für den Zeitraum vor der COVID-19-Pandemie heraus, dass der Glaube an Impfmythen neben weiteren Faktoren auch mit der Nutzung bestimmter Medienquellen assoziiert war (vgl. Stecula et al. 2020, S. 4–7). Wer traditionelle Massenmedien nutzte, hatte realistischere Vorstellungen von Impfungen und deren Nutzen und Risiken. Murphy et al. (2021, S. 3–4) wiederum stellten in einer bevölkerungsrepräsentativen Befragung für Irland und Großbritannien fest, dass impfskeptische im Vergleich zu impfbereiten Personen signifikant weniger Informationen über COVID-19 aus traditionellen Massenmedien und staatlichen Quellen bezogen. Zugleich sammelten sie jedoch signifikant mehr Informationen aus sozialen Medien. Allington et al. (2021, S. 2598–2601) fanden in verschiedenen Samples für das Vereinigte Königreich und die USA konsistente positive Zusammenhänge zwischen der Intention, sich gegen COVID-19 impfen zu lassen. und der Nutzung traditioneller Rundfunk- und Printmedien. Für soziale Medien stellten die Autor:innen in ihren Teilstudie dagegen entweder keinen oder aber einen negativen Zusammenhang mit der Impfintention fest.

Auch für Deutschland gibt es Hinweise, dass die konkrete Medien- und Informationsnutzung mit dem Impfverhalten in Verbindung stehen könnte. So gaben vor der Pandemie jeweils deutlich mehr als die Hälfte der Impfskeptiker:innen (2016: 53 %; 2018: 56 %) und mehr als jede(r) zehnte Impfbefürworter:in (2016: 11 %; 2018: 13 %) explizit an, dass sie sich in den vorangegangenen Jahren auch wegen impfkritischer Berichte in TV, Radio, Zeitungen oder Internet nicht hätten impfen lassen (vgl. Horstkötter et al. 2017, S. 76, 2019, S. 74). Im ersten Pandemie-Sommer 2020 gingen die Anteile derjenigen, die impfkritische Medienberichte als Verweigerungsgrund anführten, sowohl bei Impfskeptiker:innen (44 %) als auch bei Impfbefürworter:innen (5 %) zurück. Die großen Unterschiede zwischen den beiden Gruppen blieben jedoch bestehen (vgl. Horstkötter et al. 2021, S. 79). Gehrau et al. (2021, S. 6–8) stellten in einer bevölkerungsrepräsentativen Regionalstudie in Westdeutschland im Herbst 2020 fest, dass eine stärkere Nutzung von Social Media und alternativen Medienformaten mit einer geringeren Impfabsicht einherging, während die Nutzung von Regionalzeitungen und Informationen von Wissenschaftler:innen positiv mit der Impfintention assoziiert war. In einer weiteren deutschen Studie aus dem Herbst 2021 äußerten 56 % der ungeimpften deutschen Internetnutzer:innen, dass für sie auch die kritische Berichterstattung in den (sozialen) Medien eine Ursache war, sich bislang nicht für eine Impfung entschieden zu haben (vgl. Forsa 2021, S. 10). Mit Blick auf die Nutzung von Social Media fiel dabei insbesondere auf, dass Ungeimpfte im Vergleich zur Gesamtbevölkerung häufiger angaben, *Telegram* (38 % vs. 17 %) und *YouTube* (72 % vs. 62 %) zu nutzen (vgl. Forsa 2021, S. 36).

Was die *Intensität* der Informationssuche betrifft, vermuten einige Autor:innen, dass eine verstärkte Suche mit mehr falschem Wissen und einer geringeren Impfbereitschaft einhergeht (vgl. Betsch et al. 2019, S. 401). Mit Blick auf die COVID-19-Impfung sind die Befunde jedoch nicht eindeutig. Tatsächlich zeigten im Sommer 2021 in Deutschland diejenigen das größte Informationsbedürfnis und das intensivste Suchverhalten, die sich zu diesem Zeitpunkt noch nicht gegen COVID-19 hatten impfen lassen, jedoch einer Impfung eher aufgeschlossen gegenüberstanden oder noch unentschlossen waren (vgl. BZgA 2021, S. 19). Faasse und Newby (2020, S. 8) dagegen stellten in der Online-Befragung eines australischen Convenience-Samples fest, dass verstärkte Mediennutzung mit einer höheren (hypothetischen) Impfbereitschaft einherging, während bevölkerungsrepräsentative Befragungen in Irland und Großbritannien für die Gesamtintensität der Informationsnutzung keine Unterschiede zwischen impfskeptischen und impfbereiten Befragten zeigten (vgl. Murphy et al. 2021).

### Vertrauen in Medien und Informationsquellen

Das Vertrauen in Medien und andere Informationsquellen ist beim Impfen ein potenziell relevanter Faktor (vgl. Betsch et al. 2018, S. 17; Larson et al. 2018, S. 1600; MacDonald 2015, S. 4163; Schmid et al. 2017, S. 11; Seddig et al. 2021, S. 10; Soares et al. 2021, S. 8; Stecula et al. 2020, S. 4). Taha et al. (2013) stellten in Kanada während der H1N1-Pandemie fest, dass fehlendes Vertrauen in die Berichterstattung der Massenmedien mit einer geringeren Impfbereitschaft assoziiert war. In Großbritannien und Irland hatten Impfskeptiker im Vergleich zu Impfbefürwortern ein signifikant geringeres Vertrauen in Zeitungen, Rundfunk, Gesundheitsakteure und staatliche Stellen als Informationsquellen (vgl. Murphy et al. 2021, S. 3–4). Für die COVID-19-Impfung stellten Salmon et al. (2021, S. 2701) im Rahmen einer bevölkerungsrepräsentativen Befragung in den USA bei Gruppen mit niedrigerer Impfintention ein niedrigeres Vertrauen in Gesundheitsbehörden fest. Auch die für Deutschland repräsentativen Befunde der COSMO-Studie zeigten, dass das Vertrauen in „die Medien“ bei der COVID-19-Impfung über die verschiedenen Messzeitpunkte hinweg durchgehend hoch mit der Impfbereitschaft korrelierte (vgl. Universität Erfurt 2021). Für ihr westdeutsches Sample berichteten Gehrau et al. (2021, S. 6–8), dass ein höheres Vertrauen in bestimmte Informationsquellen wie Fernsehen, Lokalzeitungen, Wissenschaftler:innen und Gesundheitsbehörden mit einer höheren COVID-19-Impfabsicht einherging, während die Impfabsicht mit stärkerem Vertrauen in alternative Informationsquellen sank.

Aus der allgemeinen Forschung zu Medienvertrauen ist zudem bekannt, dass die Medien- und Informationsnutzung mit dem Vertrauen in Medien und Informationsquellen interagiert (vgl. Fawzi et al. 2021, S. 160–163; Newman et al. 2021, S. 11), wobei sich dieser Befund auch für das Informationsverhalten der deutschen Bevölkerung in der COVID-19-Pandemie bestätigte (vgl. Viehmann et al. 2020, S. 566). Hinweise auf kombinierte Effekte von Quellenvertrauen und Informationsnutzung auf die Impfintention fanden Gehrau et al. (2021, S. 6–7) im Rahmen ihrer Regionalstudie. Hier zeigte sich für bestimmte Informationsquellen (Fernsehen, Regionalzeitungen und Wissenschaftler:innen), denen die Befragten stärker vertrauten und die sie stärker nutzten, ein positiver Zusammenhang mit der Impfabsicht, während für andere (alternative Medien, Social Media, Familienmitglieder) ein negativer Zusammenhang mit der Impfabsicht beobachtet wurde.

Entsprechende Indizien für die COVID-19-Impfung gibt es zunehmend auch auf internationaler Ebene. So fanden etwa Zimand-Sheiner et al. (2021, S. 9–10) in einer für die israelische Bevölkerung zwischen 18 und 55 Jahren repräsentativen Studie u. a. signifikante Zusammenhänge zwischen Informationsnutzung, Medien- und Quellenvertrauen, Impfeinstellungen und Impfverhalten. Während ein höheres Vertrauen in Medien und staatliche Institutionen jeweils mit einer positiveren Einstellung gegenüber der COVID-19-Impfung einherging, die wiederum in einer höheren Impfwahrscheinlichkeit resultierte, war das Vertrauen in Social Media negativ mit der Impfeinstellung assoziiert. Zwischen der Informationsnutzung und der Impfeinstellung zeigte sich dagegen nur ein indirekter Zusammenhang, der über das Vertrauen in Institutionen und die Medien mediiert wurde.

Erste internationale Befunde zur COVID-19-Impfbereitschaft von Studierenden in den USA und verschiedenen Ländern Europas deuten darauf hin, dass auch in dieser Zielgruppe sowohl das Vertrauen in bestimmte Informationsquellen als auch die konkrete Informationsnutzung einen relevanten Einfluss auf die Impfbereitschaft haben können (vgl. Kecojevic et al. 2021; Riad et al. 2021; Tavolacci et al. 2021). In einem regionalen Sample mit 457 College-Studierenden ging verstärktes Vertrauen in Informationen zu COVID-19-Impfstoffen aus Nachrichtenmedien sowie von Regierung und Gesundheitsbehörden mit einer erhöhten Impfbereitschaft einher. Dagegen wirkte sich das Vertrauen in soziale Medien, Familie und Freunde als Informationsquellen nicht auf die Impfbereitschaft aus (vgl. Kecojevic et al. 2021, S. 1065–1066). In einer Befragung von 3089 Studierenden einer großen Universität in Frankreich äußerten sechs bzw. vier Prozent der impfskeptischen oder zögerlichen Studierenden explizit, dass es Inhalte von Massenmedien bzw. Social Media gewesen seien, die sie von einer Impfung abgebracht hätten (vgl. Tavolacci et al. 2021, S. 8). Auch in einer Befragung von 1351 Studierenden in Tschechien gaben zwölf Prozent der Befragten explizit an, sie hätten sich durch Massenmedien und Social Media in ihrer COVID-19-Impfentscheidung entscheidend beeinflussen lassen (vgl. Riad et al. 2021, S. 16–17). Die Wahrscheinlichkeit, vor einer Impfung zurückzuschrecken, lag dabei für Studierende, die Massenmedien und Social Media als Informationsquellen nutzten, deutlich höher als für Studierende, die dies nicht taten.

## Methode

Vor dem Hintergrund der Befunde scheint es plausibel, dass das Informationsverhalten auch bei Studierenden in Deutschland für die COVID-19-Impfentscheidung relevant sein könnte. Bislang, auch in internationalen Studien, galt das Interesse jedoch vor allem dem Zusammenhang mit Impfintentionen und nicht mit der tatsächlichen Impfung. Die Gruppe der Studierenden in Deutschland stand bislang ebenfalls kaum im Mittelpunkt. Die vorliegende Studie widmet sich deshalb der Bedeutung von Medien- und Informationsnutzung und von Vertrauen in Medien und andere Informationsquellen für Studierende in Deutschland bei ihrer Impfentscheidung. Dabei sollen folgende Fragen beantwortet werden:

- Inwieweit unterscheiden sich geimpfte und ungeimpfte Studierende mit hoher bzw. niedrigerer Impfintention im Hinblick auf ihre Medien- und Informationsnutzung in der COVID-19-Pandemie?
- Inwieweit unterscheiden sich geimpfte und ungeimpfte Studierende mit hoher bzw. niedrigerer Impfintention im Hinblick auf ihr Vertrauen in Medien und Informationsquellen in der COVID-19-Pandemie?

Um die Fragen zu beantworten, stützt sich die Studie auf eine Online-Befragung der Studierenden der Johannes Gutenberg-Universität (JGU) Mainz im Sommersemester 2021. Sie fand zwischen dem 21. Juni und dem 15. August 2021 statt und damit kurz nach Aufhebung der Impfpriorisierung für die COVID-19-Impfstoffe (vgl. Die Bundesregierung 2021). Die Erhebung war eingebettet in ein größeres Befragungsprojekt des interdisziplinären Verbundprojekts „Healthy Campus Mainz“, das die Studierenden der JGU im jährlichen Rhythmus zu Aspekten von Gesundheit und Studium befragt (vgl. Reichel et al. 2021). Alle eingeschriebenen Studierenden der Universität wurden in einer Vollerhebung über einen zentralen Mailverteiler angeschrieben, über den sie für gewöhnlich wichtige Nachrichten, zum Beispiel zu ihren Noten, erhalten. Als Anreiz wurden unter den Teilnehmenden u. a. Gutscheine für internationale und lokale Unternehmen im Gesamtwert von 500 € verlost. Zudem wurde beim Erreichen einer bestimmten Teilnehmendenzahl eine Spende an eine gemeinnützige Einrichtung in Aussicht gestellt.

Gegenstand der Befragung waren der *Impfstatus *(„Wurden Sie bereits gegen das Coronavirus bzw. COVID-19 geimpft?“) und die* Impfbereitschaft *(„Wie wahrscheinlich ist es, dass Sie sich impfen lassen, wenn Ihnen ein Angebot für eine Coronavirus-Impfung bzw. COVID-19-Impfung gemacht wird?“; Antwortskala, 1 = „Sehr unwahrscheinlich“, 11 = „Sehr wahrscheinlich“). Die *Intensität der allgemeinen Mediennutzung* (Fernsehen, Radio, Zeitungen und Zeitschriften, Internet) und die *Intensität der coronabezogenen Informationsnutzung* wurden in Tagen pro Woche erfasst. Die Abfrage erfolgte jeweils zweistufig (*Allgemeine Mediennutzung: 1.)*„Wie häufig nutzen Sie die folgenden Medien?“ Antwortoptionen: „Nie“, „Seltener als einmal pro Monat“, „Mindestens einmal pro Monat“, „Mindestens einmal pro Woche“, „Mindestens einmal pro Tag“; *2.)* Bei Nutzung pro Woche: „Wenn Sie nun noch einmal an eine ganz gewöhnliche Woche denken: An wie vielen Tagen dieser Woche schauen Sie fern/hören Sie Radio/lesen Sie Zeitungen & Zeitschriften/nutzen Sie das Internet?“; *Coronabezogene Informationsnutzung*: *1.) *„Wie häufig informieren Sie sich über das Thema Corona?“; Antwortoptionen: „Nie“, „Seltener als einmal pro Monat“, „Mindestens einmal pro Monat“, „Mindestens einmal pro Woche“, „Mindestens einmal pro Tag“; *2.)* Bei Nutzung pro Woche: „Wenn Sie einmal an eine ganz gewöhnliche Woche denken: An wie vielen Tagen dieser Woche informieren Sie sich über das Thema Corona?“). Zusätzlich erhoben wir, welche konkreten Informationsquellen (off- und online) die Befragten in den vorangegangenen Monaten coronabezogen genutzt hatten (z. B. *massenmediale Quellen* wie Fernsehen, Radio, Zeitungen/Zeitschriften oder Bücher, aber auch *interpersonale Quellen* wie persönliche Gespräche oder Chats mit Familienangehörigen, Freund:innen, Kolleg:innen oder Vertreter:innen der Gesundheitsberufe). Hierbei konnten die Befragten auf Angebotslisten zurückgreifen, die in früheren Erhebungen zum Gesundheitsinformationsverhalten zum Einsatz kamen und sich dort für die Abfrage als geeignet erwiesen hatten (vgl. Baumann et al. 2020; Schäfer et al. 2021). Diese Listen waren um Angebote ergänzt, die sich in der Pandemie an anderer Stelle als einflussreich andeuteten (z. B. Online-Messenger wie WhatsApp oder Telegram oder die Corona-Warn-App des RKI) und/oder für die spezielle Zielgruppe der Studierenden relevante coronabezogene Informationsquellen darstellen konnten (z. B. die Webseite der eigenen Universität).

Ferner erfragt wurden das *allgemeine* und *coronabezogene Medienvertrauen* („Ganz allgemein gefragt: Wie sehr kann man den Medien aus Ihrer Sicht in Deutschland vertrauen?“; „Wie sehr kann man den Medien beim Thema Corona vertrauen?“; jeweils 5‑stufige Antwortskala, 1 = „Überhaupt nicht“, 5 = „Voll und ganz“) sowie das *allgemeine bzw. coronabezogene Vertrauen in bestimmte Medien- und Informationsquellen* („Manche Menschen halten bestimmte Medienangebote für vertrauenswürdiger als andere. Wie ist das bei Ihnen? Bitte geben Sie an, wie vertrauenswürdig Sie die folgenden Angebote finden“; „Wie vertrauenswürdig sind die folgenden Quellen mit Blick auf das Thema Corona?“; jeweils 5‑stufige Antwortskala, 1 = „Überhaupt nicht“, 5 = „Sehr“). Als weitere Parameter zur Charakterisierung der Zielgruppe wurden das Alter in Jahren sowie das Geschlecht (männlich, weiblich, divers, offen) erhoben.

In die Auswertung kamen nach Datenbereinigung alle 1398 Fragebögen, in denen der Impfstatus klar benannt wurde. Aufgrund von Antwortoptionen, die auch die Möglichkeit beinhalteten, keine Angaben zu machen, kann die Stichprobengröße bei einzelnen Auswertungsschritten leicht variieren. In der Stichprobe waren jüngere und weibliche Studierende gemessen an der Verteilung dieser soziodemographischen Merkmale an der Universität bzw. in der Studierendenschaft in Deutschland unter den Teilnehmer:innen überrepräsentiert (Tab. 1). Letzteres entspricht einer Tendenz, die auch in anderen Studierendenbefragungen mit Gesundheitsbezug im europäischen Raum zu beobachten ist (vgl. Barello et al. 2020; Riad et al. 2021; Tavolacci et al. 2021). Dass sich zudem verstärkt Studierende mit tendenziell höherem Gesundheitsinteresse an solchen Befragungen beteiligen, lässt sich nicht ausschließen (vgl. Reichel et al. 2021).

**Table 1:** 

	Befragung SoSe 2021 (N = 1398)	Universität WiSe 2020/2021 (N = 31.194)	Deutschland WS 2020/2021 (N = 2,9 Mio.)
Alter	M = 23,7	M = 24,7	M = 23,4
Geschlecht			
Männlich	23,6 %	41,0 %	50,2 %
Weiblich	74,0 %	59,0 %	49,8 %
Divers	0,9 %	–	–
Offen	1,5 %	–	–

Mehr als zwei Drittel der befragten Studierenden hatten im Sommersemester 2021 bereits mindestens eine Impfung erhalten (69,7 %), knapp ein Drittel (30,3 %) war zu diesem Zeitpunkt noch nicht gegen COVID-19 geimpft. Damit bewegten sich die Anteile im Studierendensample ziemlich exakt auf dem Niveau, wie es im Sommer 2021 allgemeine bevölkerungsrepräsentative Befragungen für die bei Studierenden besonders relevanten Altersgruppen festgestellt hatten (vgl. BZgA 2021). Die Impfbereitschaft unter den ungeimpften Studierenden erwies sich insgesamt als hoch: Fast zwei Drittel (64,3 %) gaben an, sich „sehr wahrscheinlich“ gegen COVID-19 impfen zu lassen, knapp jede(r) Zehnte (9,3 %) äußerte, dass dies „sehr unwahrscheinlich“ sei (M = 8,9; SD = 3,4). Geimpfte Studierende (M = 23,9; SD = 5,0) waren im Mittel älter als ungeimpfte Studierende (M = 23,2; SD = 4,1), t(1392) = 2,75, *p* < 0,01. Wesentliche Geschlechterunterschiede gab es dagegen nicht.

Da trotz der Aufhebung der Impfpriorisierung die Verfügbarkeit von Impfterminen zum Zeitpunkt der Befragung im Sommer 2021 in Deutschland (und auch in Mainz) noch nicht flächendeckend gegeben war, ist es sinnvoll, die Gruppe der ungeimpften Studierenden für die Analyse noch weiter zu differenzieren. Im Folgenden unterscheiden wir daher zwischen Studierenden, die zum Zeitpunkt der Befragung noch keine Impfung erhalten, jedoch auf die Frage nach der Wahrscheinlichkeit einer Impfung im Falle eines Angebots mit „sehr wahrscheinlich“ (und damit der höchstmöglichen Ausprägung „11“) geantwortet hatten (*n* = 273), und solchen ungeimpften Studierenden, die für den Fall eines Angebots eine geringere Wahrscheinlichkeit für eine Impfung angaben (≤ „10“; *n* = 151). Hintergrund ist die Annahme, dass die beiden Gruppen zwar in ihrem Impfstatus übereinstimmen, im Hinblick auf ihre Impfbereitschaft bzw. ihr Zögern jedoch relevante Unterschiede bestehen, die sich nicht durch ein möglicherweise fehlendes Impfangebot erklären lassen. Während bei der ersten Gruppe der ungeimpften Studierenden mit hoher Impfintention zu vermuten ist, dass diese grundsätzlich zur Impfung entschlossen sind und die Impfung daher, wenn verfügbar, mit hoher Wahrscheinlichkeit in Anspruch nehmen, deutet das Antwortverhalten der ungeimpften Studierenden mit niedrigerer Impfintention auf eine insgesamt skeptische bzw. zumindest zögerlichere oder unentschlossenere Haltung gegenüber der COVID-19-Impfung hin.

Entsprechend ergeben sich zwischen den beiden Gruppen bedeutende Unterschiede bei der Impfintention („hohe Impfintention“: M = 11,0; SD = 0; „niedrigere Impfintention“: M = 5,2; SD = 3,3). Signifikante Alters- oder Geschlechterunterschiede zwischen den beiden Gruppen bestehen nicht.

Für die Vergleiche des Informationsverhaltens zwischen geimpften und ungeimpften Studierenden mit hoher und niedrigerer Impfintention wurden Chi^2^-Tests und Varianzanalysen berechnet. Da Varianzhomogenität nicht für alle interessierenden Variablen anzunehmen war, wurde auf die robustere Welch-ANOVA zurückgegriffen. Die Post-Hoc-Tests erfolgten mit dem Games-Howell Test. Um einer möglichen Alphafehler-Kumulierung entgegenzuwirken, kam mit der Bonferroni-Holm-Korrektur ein eher konservatives Verfahren zum Einsatz. Es gewährleistet, dass die Wahrscheinlichkeit für falsch-positive Befunde über alle durchgeführten Tests hinweg konstant unter *p* < 0,05 bleibt. Für die einzelnen Tests werden Unterschiede zwischen den Gruppen daher nur angenommen, wenn sich diese auf einem Signifikanzniveau von *p* ≤ 0,001 bewegen.

## Ergebnisse

### Informationsnutzung und Impfstatus

In Anbetracht ihrer möglichen Bedeutung für die Impfentscheidung stellt sich die Frage, inwieweit sich die Informations- und Mediennutzung bei geimpften und ungeimpften Studierenden mit hoher und niedrigerer Impfintention unterscheidet. Tatsächlich fällt zunächst auf, dass sich geimpfte Studierende signifikant intensiver über Corona informierten als die ungeimpften Studierenden. Es zeigten sich auch signifikante Unterschiede in der Intensität der coronabezogenen Informationsnutzung zwischen ungeimpften Studierenden mit hoher und niedrigerer Impfintention (Tab. 2). Während sich geimpfte Studierende im Mittel an vier Tagen pro Woche über Corona informierten, taten dies ungeimpfte Studierende mit hoher Impfintention im Mittel nur an 3,5 Tagen, Studierende mit niedrigerer Impfintention sogar an weniger als drei Tagen pro Woche.

**Table 2:** 

Mediennutzung/Informationsverhalten	Geimpft	Ungeimpft *hoch*	Ungeimpft *niedrig*	Welch’s F (df1; df2)	p
Fernsehnutzung (Offline)	1,9	1,4	1,7	4,30 (2; 313,67)	0,014
Radionutzung (Offline)	1,8^a^	1,2^b^	1,2^b^	9,13 (2; 334,85)*	< 0,001
Printnutzung (Offline)	1,2	0,9	0,7	4,72 (2; 333,26)	0,010
Internetnutzung	6,9	6,9	6,5	3,74 (2; 287,66)	0,025
Coronabezogene Informationsnutzung	4,0^a^	3,5^b^	2,8^c^	15,66 (2; 336,75)*	< 0,001

Welch-ANOVA (Post-Hoc-Test: Games-Howell); Gruppen mit unterschiedlichen Kennbuchstaben (a, b, c) unterscheiden sich signifikant auf dem 5 %-Niveau; Basis: 935–974 Geimpfte, 265–273 Ungeimpfte mit hoher Impfintention, 136–151 Ungeimpfte mit niedrigerer Impfintention; die angegebenen Mittelwerte basieren jeweils auf der durchschnittlichen Nutzung in Tagen pro Woche

*Unterschiede signifikant nach Bonferroni-Holm-Korrektur

Zumindest in der Tendenz zeigten geimpfte Studierende zudem im Vergleich zu den beiden Gruppen der ungeimpften Studierenden eine systematisch intensivere allgemeine Mediennutzung, wobei sich dies vor allem für Offline-Medien andeutete. Signifikante Unterschiede zwischen den Gruppen ergaben sich hier nach Bonferroni-Holm-Korrektur jedoch nur für die Radionutzung.

Hinsichtlich der konkreten Quellen, die die Studierenden zur Information über Corona nutzten, zeigten sich zum Teil deutliche Differenzen, wobei sich die allgemeine Grundtendenz systematischer Unterschiede bei der Offline-Mediennutzung zwischen ungeimpften und geimpften Studierenden bestätigten (Tab. 3). So wurden klassische TV-, Radio- und Printmedien von einem signifikant größeren Anteil der geimpften Studierenden genutzt. Zudem berichteten geimpfte Studierende in signifikant stärkerem Ausmaß von einem persönlichen Austausch mit Vertreter:innen der Gesundheitsberufe.

**Table 3:** 

Informationsquellen	Geimpft (*n* = 974)	Ungeimpft *hoch* (*n* = 273)	Ungeimpft *niedrig* (*n* = 151)	χ^2^(1)	p
Internet	95,6	93,8	90,7	6,74	0,034
Persönliche Gespräche oder Chats mit Familienangehörigen, Freund:innen, Kolleg:innen	79,4	76,2	72,8	3,92	0,141
Fernsehen (offline)	64,2	52,4	51,7	17,96*	< 0,001
Radio (offline)	46,4	33,7	31,8	21,83*	< 0,001
Zeitungen oder Zeitschriften (offline)	43,1	35,9	27,2	16,26*	< 0,001
Persönliche Gespräche oder Chats mit Ärzt:innen, Therapeut:innen, Pflegekräften	31,5	16,5	21,9	26,90*	< 0,001
Kostenlose Broschüren oder Zeitschriften von Krankenkassen, Apotheken oder anderen Anbietern (offline)	9,2	11,4	9,9	1,09	0,579
Beratungsstellen, Gesundheits- oder Bildungseinrichtungen	8,9	7,0	8,6	1,07	0,586
Bücher, Gesundheitsratgeber, Lexika	7,9	4,8	6,0	3,53	0,172
Persönliche Gespräche oder Chats mit (anderen) Patient:innen oder Betroffenen	7,5	2,9	6,0	7,44	0,024
Persönliche Gespräche oder Chats mit Apotheker:innen	4,5	2,9	2,0	3,12	0,210
Telefonische Beratungsangebote von Krankenkassen, Patienten- oder Verbraucherschutzorganisationen	2,7	2,2	1,3	1,08	0,584
Sonstige Quellen	8,7	10,6	11,3	1,61	0,446

Anteil der Studierenden in Prozent. Frage: „Wie haben Sie sich im letzten Jahr über das Thema Corona informiert?“

*Markiert sind die Informationsquellen, bei denen sich die Nutzung zwischen den Studierendengruppen nach Chi-Quadrat-Test und Bonferroni-Holm-Korrektur signifikant unterscheidet

Im Gegensatz zur Intensität der Informationsnutzung verläuft die Trennlinie bei der Art der genutzten Quellen vor allem zwischen geimpften und ungeimpften Studierenden, während sich die Muster bei ungeimpften Studierenden mit hoher und niedrigerer Impfintention tendenziell ähneln. Im Vergleich mit ihren ungeimpften Kommiliton:innen informierten sich Studierende, die zum Zeitpunkt der Befragung bereits mindestens eine Impfung gegen COVID-19 erhalten hatten, nicht nur intensiver über Corona, sondern griffen dabei auch auf ein größeres Portfolio an insbesondere traditionellen massenmedialen Quellen zurück. Sie standen zudem in deutlich stärkerem Maße in persönlichem Kontakt mit Vertreter:innen der Gesundheitsberufe. Andere Informationsquellen waren dagegen für alle Gruppen ähnlich relevant, so etwa Gespräche oder Chats mit Familienangehörigen, Freund:innen oder Kolleg:innen oder das Internet als Corona-Informationsquelle im Allgemeinen.

Sowohl für geimpfte als auch für ungeimpfte Studierende stellten Online-Angebote die mit Abstand wichtigsten Informationsquellen über Corona dar. Konkret unterschied sich die Nutzung jedoch merklich (Tab. 4). So nutzten Geimpfte in signifikant stärkerem Ausmaß soziale Medien wie *Instagram* oder *Facebook; *sie wurden nach eigenen Angaben von mehr als 51 % der geimpften und knapp 39 bzw. 40 % der ungeimpften Studierenden mit hoher bzw. niedrigerer Impfintention als Quellen herangezogen. Bei Messenger-Diensten wie *WhatsApp* oder *Telegram*, die fast jede(r) fünfte Studierende zur Information über Corona nutzte, zeigten sich dagegen keine signifikanten Unterschiede zwischen den Gruppen. Auch Videoplattformen, Suchmaschinen, Gesundheitsportale oder Webseiten von Universitäten und wissenschaftlichen Instituten frequentierten geimpfte und ungeimpfte Studierende insgesamt in ähnlich hohem Ausmaß.

**Table 4:** 

Online-Informationsquellen	Geimpft (*n* = 931)	Ungeimpft *hoch* (*n* = 256)	Ungeimpft *niedrig* (*n* = 137)	χ^2^(1)	p
Online-Nachrichtenseiten	82,3	81,3	70,1	11,51	0,003
Webseiten von Regierungen oder Behörden	77,0	78,9	63,5	13,43*	0,001
Suchmaschinen	65,0	63,3	61,3	0,84	0,658
Corona-Warn-App	59,5	51,2	14,6	97,30*	< 0,001
Social Media	51,3	38,7	40,1	16,45*	< 0,001
Webseiten von Universitäten	47,9	50,8	45,3	1,19	0,551
Videoplattformen (z. B. *YouTube*)	34,0	40,2	38,7	3,93	0,140
Online-Radio, Audio-Streaming & Podcasts	26,9	21,9	19,7	5,05	0,080
Online-Messenger (z. B. WhatsApp, Telegram)	19,3	19,1	21,2	0,28	0,869
Wikipedia oder andere Online-Lexika	15,9	16,4	13,1	0,80	0,670
Gesundheitsportale (z. B. *netdoktor, onmedia*)	14,6	14,1	13,1	0,23	0,891
Webseiten von Gesundheits- oder Patientenorganisationen	11,4	12,1	10,9	0,15	0,930
Webseiten von Ärzt:innen, Krankenhäusern, Reha- oder Pflegeeinrichtungen	13,7	15,6	17,5	1,69	0,429
Online-TV & Video-Streaming	9,3	6,3	5,1	4,58	0,101
Webseiten von Krankenkassen	8,6	9,8	8,8	0,34	0,842
Blogs zu Gesundheits- und Krankheitsthemen	6,2	6,6	9,5	2,05	0,360
Gesundheitsforen und -Communities	4,4	3,5	8,8	6,04	0,049
Online-Apotheken	3,2	2,3	5,1	2,18	0,336
Vergleichsportale zur Suche von Ärzt:innen, Krankenhäusern und Pflegeheimen	2,3	2,0	2,9	0,38	0,828
Ratgeber-Communities (z. B. *gutefrage.de*)	0,9	1,2	1,5	0,56	0,755
Medizinische Online-Beratung	1,4	0,4	2,2	2,59	0,274
Sonstige Online-Quellen	3,0	3,5	2,9	0,19	0,910

Anteil der Studierenden in Prozent. Frage: „Welche Internet-Angebote haben Sie rund um das Thema Corona genutzt?“

*Markiert sind die Informationsquellen, bei denen sich die Nutzung zwischen den Studierendengruppen nach Chi-Quadrat-Test und Bonferroni-Holm-Korrektur signifikant unterscheidet

Bemerkenswerte Unterschiede bei der Nutzung konkreter Online-Quellen ergaben sich dagegen vor allem zwischen a) den geimpften Studierenden und den ungeimpften Studierenden mit hoher Impfintention auf der einen und b) den ungeimpften Studierenden mit niedrigerer Impfintention auf der anderen Seite. So nutzten Letztere in signifikant geringerem Ausmaß offizielle Webseiten von Regierungen oder Behörden, während zwischen geimpften Studierenden und ungeimpften Studierenden mit hoher Impfintention kaum Unterschiede zu beobachten waren. Ähnliche Tendenzen deuteten sich zudem bei der Nutzung von journalistischen Online-Nachrichtenseiten an. Auch die offizielle Corona-Warn-App, die vom Robert Koch-Institut (RKI) kostenfrei zur Verfügung gestellt wurde, nutzten mit knapp 60 % der geimpften und mehr als 51 % der ungeimpften Studierenden mit hoher Impfintention jeweils mehr als die Hälfte der beiden Studierendengruppen, aber weniger als jeder Achte der ungeimpften Studierenden mit niedrigerer Impfintention. Tatsächlich könnte sich hier im Nutzungsverhalten bemerkbar machen, was im Hinblick auf die Impfbereitschaft, aber auch die allgemeine Akzeptanz coronabezogener Maßnahmen in der COVID-19-Pandemie auch in Deutschland immer wieder sichtbar wurde (vgl. u. a. BZgA 2021; Gehrau et al. 2021): die Bedeutung des Vertrauens in staatliche Institutionen und traditionelle Medien.

### Quellen- und Medienvertrauen und Impfstatus

In der Tendenz zeigte sich über alle Bereiche hinweg, dass ungeimpfte Studierende mit geringerer Impfintention staatlichen und gesellschaftlichen Institutionen als Informationsquellen über Corona im Mittel systematisch weniger Vertrauen entgegenbrachten als geimpfte Studierende und Studierende mit hoher Impfintention (Tab. 5). Besonders deutlich fielen die Unterschiede im Hinblick auf solche Institutionen aus, die mit Empfehlungen und politischen Maßnahmen den Kurs der Pandemiebekämpfung in Deutschland öffentlich mitprägten. So ergaben sich signifikante Unterschiede im Vertrauen nach Bonferroni-Holm-Korrektur für die Bundes- und die Landesregierung, die Stadt, politische Parteien sowie für zentrale Gesundheitsbehörden und -organisationen auf regionaler (Gesundheitsämter), nationaler (RKI, Ständige Impfkommission [STIKO]) und internationaler Ebene (WHO). Aber auch in den Ethikrat, der sich in der Pandemie immer wieder mit Stellungnahmen u. a. zur Impfpflicht (vgl. Deutscher Ethikrat 2021) öffentlich positionierte, ebenso wie in Universitäten und wissenschaftliche Institute, Gewerkschaften und sogar die eigene Universität hatten ungeimpfte Studierende mit niedrigerer Impfintention nach eigenen Angaben ein signifikant geringeres Vertrauen. Ähnliche systematische Tendenzen waren darüber hinaus auch im Hinblick auf Institutionen und Repräsentant:innen des Gesundheitssystems wie Ärzt:innen, Apotheker:innen, Pfleger:innen, Krankenhäusern und Krankenkassen zu beobachten, denen die ungeimpften Studierenden mit geringerer Impfintention signifikant weniger vertrauten.

**Table 5:** 

Vertrauen in	Geimpft	Ungeimpft *hoch*	Ungeimpft *niedrig*	Welch’s F (df1; df2)	p
*Regierungen & Gesundheitsbehörden (Inland)*					
Bundesregierung	3,57^a^	3,50^a^	2,76^b^	31,88 (2; 301,94)*	< 0,001
Landesregierung	3,61^a^	3,63^a^	2,88^b^	34,22 (2; 309,49)*	< 0,001
Stadt	3,67^a^	3,67^a^	3,14^b^	21,30 (2; 310,78)*	< 0,001
Robert Koch Institut (RKI)	4,58^a^	4,70^b^	3,59^c^	50,20 (2; 303,11)*	< 0,001
Ständige Impfkommission (STIKO)	4,31^a^	4,26^a^	3,27^b^	51,28 (2; 299,88)*	< 0,001
Gesundheitsämter	3,99^a^	4,09^a^	3,28^b^	26,20 (2; 301,77)*	< 0,001
*Regierungen & Gesundheitsbehörden (Ausland)*					
Regierungen und Behörden Ausland	2,88	2,95	2,74	2,94 (2; 300,91)	0,054
Weltgesundheitsorganisation (WHO)	4,37^a^	4,39^a^	3,54^b^	34,28 (2; 295,38)*	< 0,001
*Gesundheitssystem & Gesundheitsberufe*					
Ärzt:innen	4,31^a^	4,44^b^	3,92^c^	17,60 (2; 313,70)*	< 0,001
Apotheker:innen	4,03^a^	4,12^a^	3,59^b^	16,69 (2; 305,49)*	< 0,001
Pfleger:innen	3,89^a^	4,06^b^	3,68^c^	8,46 (2; 312,63)*	< 0,001
Krankenhäuser	4,15^a^	4,21^a^	3,64^b^	16,88 (2; 303,02)*	< 0,001
Krankenkassen	3,61^a^	3,74^a^	3,12^b^	16,78 (2; 298,38)*	< 0,001
*Wissenschaft & Gesellschaft: Institutionen*					
Universitäten und wissenschaftliche Institute	4,40^a^	4,48^a^	4,01^b^	12,99 (2; 307,51)*	< 0,001
Eigene Universität	4,11^a^	4,20^a^	3,63^b^	18,34 (2; 304,65)*	< 0,001
Ethikrat	3,66^a^	3,50^b^	3,06^c^	19,67 (2; 311,38)*	< 0,001
Gewerkschaften	3,02^a^	3,09^a^	2,77^b^	7,66 (2; 314,73)*	0,001
Wirtschafts- und Industrieverbände	2,65	2,53	2,51	2,81 (2; 312,10)	0,062
Politische Parteien	2,69^a^	2,67^a^	2,24^b^	17,08 (2; 313,02)*	< 0,001
Kirchen	2,26	2,08	2,15	3,43 (2; 315,41)	0,033
*Wissenschaft & Gesellschaft: Einzelpersonen*					
Einzelne Wissenschaftler:innen	3,61	3,64	3,44	2,37 (2; 308,69)	0,095
Einzelne Politiker:innen	2,69	2,66	2,41	5,38 (2; 299,05)	0,005
Gewöhnliche Menschen *(persönlich bekannt)*	3,05	2,94	3,11	2,17 (2; 312,36)	0,116
Gewöhnliche Menschen *(nicht persönlich bekannt)*	2,05	1,98	2,16	2,18 (2; 320,47)	0,114

Welch-ANOVA (Post-Hoc-Test: Games-Howell); Gruppen mit unterschiedlichen Kennbuchstaben (a, b, c) unterscheiden sich signifikant auf dem 5 %-Niveau; Basis: 939–944 Geimpfte, 264–266 Ungeimpfte mit hoher Impfintention, 135–137 Ungeimpfte mit niedrigerer Impfintention; Frage: „Wie vertrauenswürdig sind die folgenden Quellen mit Blick auf das Thema Corona?“; 5‑stufige Antwort-Skala von 1 = „überhaupt nicht“ bis 5 = „sehr“

*Unterschiede signifikant nach Bonferroni-Holm-Korrektur

Besonders bemerkenswert ist, dass dagegen die ungeimpften Studierenden mit *hoher* Impfintention den Institutionen und Individuen in aller Regel ein vergleichbar hohes bzw. niedriges Vertrauen entgegenbrachten wie die Studierenden, die sich bereits gegen COVID-19 hatten impfen lassen. Zum Teil hatten sie in bestimmte Institutionen wie RKI und Gesundheitspersonal sogar mehr Vertrauen. Dies lässt vermuten, dass der Impfstatus zum Zeitpunkt der Befragung eher weniger mit dem Ausmaß des Vetrauens in Verbindung stand.

Diese speziellen Beobachtungen für die Gruppe der ungeimpften Studierenden mit hoher Impfintention und auch die beobachteten Vertrauensunterschiede zwischen den Gruppen setzten sich beim Medienvertrauen fort. Ungeimpfte Studierende mit niedrigerer Impfintention bekannten hier ein signifikant niedrigeres allgemeines und coronabezogenes Vertrauen in die Medien in Deutschland (Tab. 6). Interessanterweise äußerten alle drei Studierendengruppen mehr coronabezogenes als allgemeines Medienvertrauen, wobei sich die absoluten Werte hier um den Skalenmittelpunkt bewegten. Das Vertrauen in einzelne Medienangebote variierte in Abhängigkeit von den Quellen deutlich, wobei die Studierenden gruppenübergreifend den Angeboten des öffentlich-rechtlicher Rundfunks, der überregionalen und der regionalen Zeitungen das stärkste (und auch gemessen an der Skala: ein überdurchschnittliches) Vertrauen entgegenbrachten. Während das Vertrauen von geimpften und ungeimpften Studierenden in Boulevardzeitungen, Online-Portale, Videoplattformen oder Messenger-Dienste ähnlich (niedrig) ausfiel, brachten ungeimpfte Studierende mit niedrigerer Impfintention Angeboten des öffentlich-rechtlichen und privaten Rundfunks sowie überregionaler und regionaler Zeitungen signifikant weniger Vertrauen entgegen. Alternative Nachrichtenseiten und Blogs dagegen genossen bei dieser Gruppe ein signifikant höheres Vertrauen als bei den Kommiliton:innen, wobei zu beachten ist, dass das absolute Vertrauensniveau auch hier bei allen Studierendengruppen deutlich schwächer ausgeprägt war als bei den meisten traditionellen Medienangeboten.

**Table 6:** 

Vertrauen in	Geimpft	Ungeimpft *hoch*	Ungeimpft *niedrig*	Welch’s F (df1; df2)	p
*Globales Vertrauen in Medien*					
Allgemeines Medienvertrauen	2,89^a^	2,99^a^	2,37^b^	21,11 (2; 319,19)*	< 0,001
Coronabezogenes Medienvertrauen	3,32^a^	3,43^a^	2,67^b^	35,18 (2; 316,72)*	< 0,001
*Vertrauen in bestimmte Medienquellen*					
Öffentlich-rechtlicher Rundfunk (inkl. Online-Angebote)	4,39^a^	4,43^a^	3,53^b^	37,06 (2; 307,58)*	< 0,001
Privater Rundfunk (inkl. Online-Angebote)	3,02^a^	3,00^a^	2,60^b^	12,38 (2; 319,64)*	< 0,001
Überregionale Zeitungen (inkl. Online-Angebote)	4,13^a^	4,12^a^	3,64^b^	14,98 (2; 315,56)*	< 0,001
Regionalzeitungen (inkl. Online-Angebote)	3,70^a^	3,76^a^	3,31^b^	15,63 (2; 327,44)*	< 0,001
Boulevardzeitungen (inkl. Online-Angebote)	1,70	1,63	1,79	2,02 (2; 319,31)	0,135
Online-Portale (z. B. *t‑online.de, gmx.de*)	2,47	2,52	2,44	0,60 (2; 319,97)	0,552
Alternative Nachrichtenseiten und Blogs	2,10^a^	2,17^a^	2,57^b^	15,72 (2; 324,33)*	< 0,001
Videoplattformen (z. B. *YouTube*)	2,55	2,57	2,68	1,46 (2; 323,00)	0,234
Soziale Netzwerke (z. B. *Facebook, Instagram*)	2,15^a^	1,96^b^	2,01^ab^	7,29 (2; 329,52)*	0,001
Messenger-Dienste (z. B. *WhatsApp, Telegram*)	1,68	1,63	1,88	4,38 (2; 318,81)	0,013

Welch-ANOVA (Post-Hoc-Test: Games-Howell); Gruppen mit unterschiedlichen Kennbuchstaben (a, b) unterscheiden sich signifikant auf dem 5 %-Niveau; Basis: 944–959 Geimpfte, 265–268 Ungeimpfte mit hoher Impfintention, 139–144 Ungeimpfte mit niedrigerer Impfintention; Vertrauens-Skalen jeweils von 1 = „überhaupt nicht“ bis 5 = „voll und ganz“

*Unterschiede signifikant nach Bonferroni-Holm-Korrektur

Letzteres galt auch für das Vertrauen in Social Media-Angebote wie *Instagram* oder *Facebook*, denen allerdings geimpfte Studierende auf insgesamt niedrigem Niveau signifikant mehr vertrauten als ungeimpfte Studierende mit hoher Impfintention.

## Fazit

Die Frage, wer sich weshalb (nicht) gegen COVID-19 impfen lässt, war in der COVID-19-Pandemie auch in Deutschland aufgrund ihrer Relevanz für die öffentliche Gesundheit von großem Interesse. Studierende sind hier eine wichtige Zielgruppe. Mit einer Online-Befragung sind wir vertrauens- und informationsbezogenen Unterschieden zwischen ungeimpften und geimpften Studierenden nachgegangen. Hierbei zeigte sich, dass sich geimpfte und ungeimpfte Studierende mit hoher und niedrigerer Impfintention im Sommer 2021 im Hinblick auf ihr Informationsverhalten zum Teil deutlich voneinander unterschieden. Geimpfte Studierende informierten sich intensiver über Corona als ungeimpfte Studierende, insbesondere als solche mit niedrigerer Impfintention, und zogen hierfür ein breiteres Portfolio an Informationsquellen, insbesondere an traditionellen Offline-Medien, heran als ihre ungeimpften Kommiliton:innen. Letztere bezogen zudem auch seltener Corona-Informationen aus einem persönlichen Austausch mit Vertreter:innen der Gesundheitsberufe.

Unsere Ergebnisse legen jedoch nahe, dass der Impfstatus der Studierenden allein im Sommersemester 2021 für die Betrachtung eines möglichen Zusammenhangs mit dem Informationsverhalten nicht ausreicht. Tatsächlich lassen sich innerhalb der Gruppe der ungeimpften Studierenden mit Blick auf die Impfintention zwei Subgruppen identifizieren, die sich auch im Hinblick auf ihre Informations- und Mediennutzung und ihr Vertrauen in Informations- und Medienquellen systematisch voneinander unterscheiden: ungeimpfte Studierende mit hoher und ungeimpfte Studierende mit niedrigerer Impfintention. Dabei lohnt sich ein spezieller Blick auf die Gruppe derjenigen, die sich bis zum Zeitpunkt der Befragung noch nicht hatten impfen lassen, jedoch angaben, dies im Falle eines Angebots mit sehr hoher Wahrscheinlichkeit zu tun. Denn tatsächlich deutet sich hier in den Daten an, dass es nicht nur das fehlende Impfangebot und die schwierige Lage bei der Vergabe der Impftermine im Sommersemester 2021 gewesen sein könnten, die bei ihnen eine Impfung trotz hoher Impfintention erschwert hatten. Vielmehr existierten auch kommunikationsbezogene Faktoren, die diesen Impfstatus begünstigt haben könnten. So informierte sich auch diese Gruppe im Vergleich zu den geimpften Studierenden signifikant weniger über Corona und nutzte für solche Informationen seltener klassische Massenmedien oder Kontakte zu Fachvertreter:innen wie z. B. Ärzt:innen. Und auch sonst deutet vieles in der Quellennutzung darauf hin, dass diese Studierenden ein deutlich eingeschränkteres Portfolio an coronabezogenen Informationsangeboten in Anspruch nahmen. Es scheint daher nicht ganz abwegig, dass sich der Impfstatus dieser Gruppe trotz hoher Impfintention u. a. durch ein Zusammenspiel zwischen den faktischen Gegebenheiten – Mangel an (für die Zielgruppe geeigneten) Impfstoffen, fehlende Termine – und einer individuell zurückhaltenderen Informationsnutzung erklärt. So könnten etwa kurzfristig verfügbare Impftermine oder -aktionen oder andere wichtige Informationen, die nahelegen, wie man kurzfristig einen Impftermin ergattert, trotz hoher Intention an dieser Studierendengruppe vorbeigegangen sein, weil sie die entsprechenden Informationen aufgrund des eingeschränkteren Kontakts mit Massenmedien und Gatekeepern im Gesundheitssystem nicht erhalten haben.

Fehlendes Vertrauen in die Corona-Informationen von Massenmedien, staatlichen, medizinischen oder gesellschaftlichen Institutionen dürfte bei dieser speziellen Gruppe dagegen im Hinblick auf die Impfung eher keine Rolle gespielt haben. Es bewegte sich bei ungeimpften Studierenden mit hoher Impfintention durchgehend auf ähnlichem Niveau oder war sogar noch stärker ausgeprägt als das der geimpften Kommiliton:innen. Dies war bei der Gruppe der ungeimpften Studierenden mit niedrigerer Impfintention anders.

Diese spezielle Gruppe der ungeimpften Studierenden war zunächst dadurch gekennzeichnet, dass sie äußerten, ein Impfangebot nicht mit maximal hoher Wahrscheinlichkeit annehmen zu wollen, wenn es ihnen angeboten würde. Es handelt sich also hier um „Impfzögerer“ im engeren Sinne. Diese Studierenden nutzten im Vergleich mit geimpften Studierenden, aber auch ihren ungeimpften Kommiliton:innen mit hoher Impfintention signifikant weniger intensiv Informationen über Corona. Wenn, dann ähnelten ihre Quellen zwar in den meisten Bereichen denen der übrigen ungeimpften Studierenden stark, jedoch mieden sie offizielle Webseiten von Regierungen und Behörden, die offizielle Corona-Warn-App oder auch mit Einschränkungen journalistische Nachrichtenseiten signifikant häufiger. Sie zeigten darüber hinaus ein signifikant geringeres allgemeines und coronabezogenes Medienvertrauen und misstrauten traditionellen Angeboten des öffentlich-rechtlichen und privaten Rundfunks sowie traditionellen Printmedien und ihren Online-Ablegern mehr als geimpfte und ungeimpfte Kommiliton:innen mit hoher Impfintention. Alternative Nachrichtenseiten und Blogs schätzten sie dagegen, wenn auch ebenfalls unterdurchschnittlich, so doch signifikant vertrauenswürdiger ein als ihre Mitstudierenden. Ein systematisch geringeres Vertrauen war bei ihnen zudem in staatliche und medizinische Institutionen und Akteur:innen zu beobachten.

Die Befunde stehen damit ganz überwiegend in Einklang mit internationalen Erkenntnissen, sowohl der Medienvertrauensforschung (vgl. u. a. Fawzi et al. 2021; Jakobs et al. 2021) als auch der Forschung zu kommunikationsbezogenen Ursachen des Nicht-Impfens. Auch in unserer Untersuchung war es so, dass impfzögerliche Studierende medienskeptischer waren und Informationen aus offiziellen Quellen weniger stark vertrauten.

Unsere Daten bestätigen zudem Erkenntnisse, wonach ein größeres Vertrauen in alternative Nachrichtenformate mit zögerlichem Impfverhalten einhergehen könnte. Dass sich dies auch bei einer tendenziell homogenen und hochgebildeten Zielgruppe wie Universitätsstudierenden zeigt, macht deutlich, wie wichtig es ist, sich in Zukunft mit den entsprechenden Wechselbeziehungen auch mit Blick auf die allgemeine Bevölkerung noch stärker auseinanderzusetzen. In Anbetracht der systematischen Unterschiede ließe sich vermuten, dass diese Tendenzen (auch) mit den Inhalten zu tun haben könnten, die in diesen Medienformaten zu COVID-19 und der COVID-19-Impfung vermittelt wurden und werden. Denkbar wäre jedoch auch, dass Personen mit bestimmten Wahrnehmungen und Einstellungen gegenüber der COVID-19-Impfung gezielt auf Angebote zurückgriffen, die zu ihrer eigenen Haltung passten und denen sie stärker vertrauten. Um diese Annahmen empirisch zu prüfen, wären längsschnittliche Verknüpfungen von Inhaltsanalysedaten und Befragungsdaten notwendig, die uns aber leider nicht vorliegen.

Gleichzeitig widersprechen unsere Daten internationalen Beobachtungen, wonach eine gesteigerte Social Media-Nutzung negativ mit der Impfbereitschaft assoziiert ist (vgl. u. a. Gehrau et al. 2021; Riad et al. 2021; Zimand-Sheiner et al. 2021). Tatsächlich war bei den von uns befragten Studierenden das Gegenteil der Fall: Geimpfte Studierende gaben sowohl ein signifikant höheres Maß an Vertrauen in soziale Medien als Nachrichtenquellen an als auch eine signifikant intensivere Nutzung von Corona-Informationen in den sozialen Medien. Zu beachten ist jedoch, dass sich das absolute Vertrauen in Social Media-Angebote bei allen Gruppen im unteren bis mittleren Bereich bewegte.

Da bevölkerungsrepräsentative Studien in anderen Ländern in der Vergangenheit weder positive noch negative Zusammenhänge zwischen Social Media-Nutzung und Impfbereitschaft feststellten (vgl. u. a. Murphy et al. 2021), machen diese heterogenen Befunde aus unserer Sicht vor allem die enorme Komplexität deutlich, die das veränderte Gesundheitsinformationsverhalten mit sich bringt. Denn insgesamt scheint sich auch in unserem Sample der allgemeine Trend niederzuschlagen, dass Social Media-Kanäle von jüngeren Erwachsenen verstärkt auch bei gesundheits- und impfbezogen Themen als Informationsquellen in Betracht gezogen werden (vgl. Horstkötter et al. 2019, 2021). Egal ob impfkritisch oder impfbefürwortend: Studierende informieren sich ganz überwiegend online – und sie nutzen hierfür auch (mehr oder weniger selbstverständlich) soziale Medien. Dabei gilt es gerade bei diesen jungen Zielgruppen ganz besonders zu beachten, dass die Nutzung von Kanälen allein noch nichts über die konkret genutzten Inhalte und deren Qualität aussagt. So ist nicht auszuschließen, dass geimpfte Studierende auf Social Media-Kanälen in den letzten Monaten verstärkt mit impfbefürwortenden Inhalten in Berührung gekommen sind, während ungeimpfte Studierende mit niedrigerer Impfintention verstärkt impfkritischeren Inhalten ausgesetzt gewesen sein könnten.

Auch um dieser Frage nachzugehen, wäre eine tiefergehende Analyse notwendig, die auf Grundlage unserer Daten leider nicht möglich ist. Unsere Studie weist zudem naturgemäß weitere Limitationen auf, die u. a. die Stichprobe betreffen. So wurden zum einen nur Studierende einer westdeutschen Universität befragt, zum anderen weicht die Zusammensetzung des Samples trotz des Versuchs einer Vollerhebung hinsichtlich Alter und Geschlecht von der Grundgesamtheit der Studierenden in Deutschland und der befragten Universitätsstudierenden im Speziellen ab. Da es den Studierenden freistand, an der Befragung teilzunehmen, ist Selbstselektion ein relevanter Faktor, der sich beispielsweise in einem tendenziell höheren Themeninteresse der Teilnehmer:innen im Vergleich mit der allgemeinen Studierendenschaft äußern könnte. Dies gilt es bei der Interpretation zu beachten. Gleichwohl stimmen die Anteile geimpfter und ungeimpfter Studierender im Sample ziemlich genau mit den entsprechenden Anteilen in den relevanten Altersgruppen in bevölkerungsrepräsentativen Befragungen überein, die zu diesem Zeitpunkt in Deutschland stattfanden (vgl. BZgA 2021). Dies lässt darauf schließen, dass mit Blick auf das Impfverhalten trotz der Einschränkungen durchaus valide Rückschlüsse möglich sind.

Gewisse Limitationen in der Fragebogengestaltung ergaben sich zudem durch das spezielle Studiendesign. Wie oben dargelegt, war die Studie in eine größere, jährlich wiederkehrende Erhebung zur psychischen und physischen Gesundheit und den Studienbedingungen eingebettet. Dies ermöglichte zwar die Kontaktaufnahme mit sämtlichen eingeschriebenen Studierenden der Universität, führte jedoch auch dazu, dass der zur Verfügung stehende Platz für impf- und kommunikationsspezifische Items insgesamt deutlich begrenzt war. So wurde etwa die Mediennutzung für alle Kanäle in Tagen pro Woche erfasst, obwohl eine noch detailliertere Aufschlüsselung der Internetnutzung in Minuten pro Tag und/oder getrennt für bestimmte Online-Kanäle ein noch umfassenderes Bild ermöglicht hätte. Auch wurde die Nutzung bestimmter Quellen lediglich dichotom für einen festgelegten Zeitraum abgefragt; ein feinerer Detailgrad der jeweiligen Nutzungsintensität hätte hier weitreichendere Aussagen erlaubt. Zu problematisieren ist sicher auch die Abfrage der Informationsquellen mit Hilfe recht globaler Kategorien (z. B. Social Media oder Online Messenger anstelle von „*Facebook*“, „*Instagram*“, „*TikTok*“ bzw. „*WhatsApp*“ oder „*Telegram*“). Eine feiner differenzierte Liste an Angeboten könnte natürlich mehr Rückschlüsse erlauben, wenn etwa feststellbar wäre, dass sich ungeimpfte Studierende mit hoher Impfintention vornehmlich auf den Social Media-Kanälen der öffentlich-rechtlichen Rundfunkanstalten informiert hätten, während ungeimpfte Studierende mit niedriger Impfintention vor allem alternative Quellen rezipierten.

Zu berücksichtigen ist zudem, dass das gewählte Verfahren der Bonferroni-Holm-Korrektur, das einer möglichen Alphafehlerkumulierung entgegenwirken soll, ein sehr konservatives Verfahren ist. Aufgrund der vorgenommenen Korrektur, die dazu führt, dass im vorliegenden Fall Unterschiede erst ab einem Niveau von 0,001 als signifikant angenommen werden, kann es sein, dass weitere signifikante Unterschiede zwischen den Gruppen übersehen wurden. Wir haben uns hier in der Tendenz für eine konsequente Minimierung des Alphafehlers entschieden, wissend, dass Betafehler nicht ausgeschlossen sind.

Gleichwohl sind unsere Befunde insgesamt ein deutlicher Hinweis darauf, dass medien- und quellenbezogene Vertrauensaspekte und das Informationsverhalten bei der COVID-19-Impfentscheidung von Studierenden eine relevante Rolle spielen. Die Studie liefert zudem wichtige Erkenntnisse zum studentischen Impfverhalten und möglichen kommunikationsbezogenen Einflussfaktoren, die auch und insbesondere bei der Konzeption geeigneter zielgruppengerechter Kampagnen zur Steigerung der Impfbereitschaft nützlich sein könnten (vgl. Rossmann 2010).

Grundsätzlich scheint es sinnvoll, Studierende dabei zu unterstützen, bei ihrer Informationssuche die Qualität der dargebotenen Quellen noch besser beurteilen zu können. Neben spezifischen Maßnahmen gegen die Verbreitung unseriöser Informationen kann es sich hier insbesondere lohnen, die Medien- und Gesundheitskompetenz der Studierenden mit gezielten Programmen zu stärken. So könnten sie noch besser in die Lage versetzt werden, unabhängig von den genutzten Kanälen zwischen tendenziell seriösen und unseriösen Anbietern und Quellen zu unterscheiden und auf dieser Grundlage für sich adäquate Gesundheits- und Impfentscheidungen zu treffen. Sinnvoll könnte zudem sein, bei der Vermittlung von impfbezogenen Informationen verstärkt auf Kanäle und Akteure zu setzen, die bei der Zielgruppe insgesamt und speziell bei den Impfskeptikern ein hohes Maß an Vertrauen genießen. In unserer Befragung zeigte sich, trotz der beobachteten deutlichen Unterschiede zwischen den Gruppen, dass selbst die Studierenden mit geringerer Impfintention Akteur:innen des Gesundheitssystems, dem RKI und auch Universitäten und anderen wissenschaftlichen Instituten als Informationsquellen immer noch vergleichsweise stark vertrauten. Hier könnte sich die Sozialisation im akademischem Umfeld und eine grundsätzliche Offenheit für wissenschaftliche Erkenntnisse bemerkbar machen, was bei der Ansprache dieser speziellen Zielgruppe in Kampagnen genutzt werden könnte. Auffällig ist in diesem Zusammenhang zudem, dass ein deutlich geringerer Anteil der ungeimpften Studierenden in der Befragung angab, sich mit Vertreter:innen der Gesundheitsberufe ausgetauscht zu haben. Daher könnte es hilfreich sein, Studierende noch stärker ins Gespräch mit Gesundheitsexper:innen zu bringen und dabei auch die Universitäten als Arbeits- und Lebenswelten von Studierenden in den Impfkampagnen stärker einzubeziehen.

## Footnotes

1 Unter Impfmüdigkeit wird die verzögerte Akzeptanz oder Ablehnung einer Impfung trotz ihrer Verfügbarkeit verstanden (vgl. Betsch et al. 2019; MacDonald 2015).
